# Innovative Technology Based Interventions for Psychological Treatment of Common Mental Disorders

**DOI:** 10.3390/jcm9103075

**Published:** 2020-09-24

**Authors:** Tara Donker, Annet Kleiboer

**Affiliations:** 1 Department of Clinical, Neuro and Developmental Psychology, Section Clinical Psychology, Vrije Universiteit Amsterdam, Van der Boechorststraat 1, 1081 BT Amsterdam, The Netherlands; a.m.kleiboer@vu.nl; 2 Amsterdam Public Health Research Institute Amsterdam, Van der Boechorststraat 7, 1081 BT Amsterdam, The Netherlands; 3 Department of Psychology, Laboratory of Biological and Personality Psychology, Albert Ludwigs-University of Freiburg, Peter-Kaplan Meierstrasse 8, 79104 Freiburg im Breisgau, Germany

The present Special Issue of *Journal of Clinical Medicine* includes a series of important papers that aim to further the evidence base of innovative technological advances in the screening and treatment of mental health, and to further our understanding of their implications for mental health care.

The article by Colombo et al. [1] provides a systematic review of technology-based ecological momentary assessment (EMA) and ecological momentary intervention (EMI) for Major Depressive Disorder (MDD). EMA refers to assessments that take place in the environment of the participant close to the time of the experience. EMI refers to interventions in the environment of the participants close to the time of the experience. Their systematic review identified 32 studies using EMA for the assessment of MDD and eight studies targeting EMI. The authors concluded that the widespread adoption of EMA for the investigation of MDD has led to novel insights into different aspects of the disease, including emotion reactivity, cortisol patterns and daily rumination. This review found only four EMIs for depression, two of which were tested in a randomized controlled trial (RCT). Although results seem encouraging, more high-quality EMI trials are needed as well as improving individual tailoring and engagement of EMAs and EMIs. Furthermore, the gap between research and clinical practice is quite wide, as evidenced by the low number of studies in clinical settings. Elaborating on this topic, the paper of Genugten et al. [2] investigated the experienced burden of and adherence to EMA in persons with current affective disorders; remitted persons; and healthy controls. Results demonstrated that EMAs are slightly more burdensome to persons with affective disorders but that that does not impact adherence. They concluded that EMA is feasible to apply to persons with affective disorders.

The paper by Titov et al. [3] provides 10 lessons that the authors have learned while establishing and delivering internet-delivered interventions through Digital Mental Health Services (DMHS) as part of routine care. With their findings they anticipated that these lessons would help those launching similar clinics. The authors learned that DMHS can improve access to care for those who really need care, that DMHS deliver more than treatment services (namely, information and assessment services) and that DMHS are used by a broad-cross-section of the population. Furthermore, it is important that robust systems for therapist training are in place and supervision of therapists is essential. Additionally, specialist skills to operate DMHS are required (e.g., developing expertise in the evaluation of risk via telephone or online communication). The authors further learned about the importance of external-facing activities to overcome challenges of integrating DMHS within health systems (e.g., the challenging complexity of health systems and their resistance to change) and that DMHS can inform future mental health policy to help improve the broader mental health system by presenting data drawn from a broad cross section of the community. Despite the challenges, the authors are highly optimistic about the potential of DMHS to reduce the global burden of the high prevalence of mental health disorders. Elaborating on dissemination of Internet-delivered treatment in routine care, the paper of Mol et al. [4] describes a qualitative study targeting therapists’ perspectives of blended Internet-delivered interventions, specifically, cognitive behavioural therapy (CBT). In blended Internet-delivered interventions, face-to-face treatment with a therapist was combined with online therapy. The results demonstrated that therapists were positive about blended CBT (bCBT) and that high uptake was expected but that therapists did not experience time-savings—rather the opposite. In line with Titov et al. [3], they also reported that training therapists is very important to overcome barriers of Internet-delivered CBT uptake. Challenges that were identified included technical issues and a difficulty integrating bCBT in daily life. bCBT was also the focus of research in the paper by Kooistra et al. [5]. In this paper, results from an RCT in which the working alliance in bCBT vs. face-to-face CBT for depression in specialized mental health were reported. The authors demonstrated that working alliance ratings were high in both groups by both patients and therapists. This means that replacing a proportion of the face-to-face sessions with online sessions and online therapist feedback did not have a negative effect on working alliance and treatment effect. The authors noted that in the face-to-face CBT condition but not in bCBT, lower depression scores were associated with higher alliance ratings. They concluded that the online component of bCBT may have led patients to evaluate the working alliance differently from patients receiving face-to-face CBT only.

In the paper by Friedl et al. [6], the authors investigated what the most important predictors are in determining optimal treatment allocation to treatment as usual or blended treatment. Furthermore, they investigated if model-determined treatment allocation using this predictive information and the personalized advantage index (PAI) approach would result in better outcomes. Using data from an RCT comparing efficacy of treatment as usual and blended treatment in depressive outpatients, they demonstrated that two prognostic predictors, namely, pre-treatment symptomatology and treatment expectancy, influence optimal treatment allocation but that this needs to be tested empirically. Furthermore, the results also showed an advantage of model-determined treatment allocation. One-third of the patients had a PAI larger than 5, meaning they would have improved significantly if they had received their “optimal” treatment. 

In the paper of Moser et al. [7], the authors investigated an Internet-delivered self-help treatment targeting adjustment problems. The authors used an RCT design among *n* = 98 participants that were randomly assigned to care as usual (CAU) or CAU plus the online intervention. Results demonstrated a comparable reduction of symptom burden in both groups, while significantly fewer depressive symptoms and significantly higher quality of life were demonstrated in the experimental group. The usability of the intervention was rated above average. These results suggest that their intervention “Back to your own life” (German acronym: ZIEL), may contribute to the treatment of Adjustment Disorder by means of a scalable low-barrier approach. 

Finally, in the paper by Donker et al. [8], user engagement with a self-guided app-based virtual reality (VR) CBT for acrophobia symptoms was examined. Results demonstrated that the majority of participants continued to finish all VR levels and that self-reported fear consistently decreased between the start and finish of level. The authors suggest that it might be more beneficial to play one level for a longer time period instead of practicing many VR levels. Most participants progressed effectively to the highest self-exposure level, despite the absence of a therapist.

All in all, results from the papers in this Special Issue show that technological interventions can increase the scalability and dissemination of treatment for patients in need, and that they do not affect treatment outcome or working alliance, although there are many barriers to overcome, such as training of therapists, technical problems and complex health care systems. More research is needed to investigate such interventions in naturalistic settings.
